# Solid Papillary Carcinoma of the Breast With Invasion and Aggressive Characteristics: A Case Report

**DOI:** 10.7759/cureus.106079

**Published:** 2026-03-29

**Authors:** Yuto Kosaka, Shoji Oura

**Affiliations:** 1 Department of Surgery, Kishiwada Tokushukai Hospital, Kishiwada, JPN

**Keywords:** breast cancer, linear calcifications not pointing toward the nipple, small cysts, solid papillary carcinoma with invasion, weak high signals on t2-weighted images

## Abstract

Solid papillary carcinoma (SPC) is a rare disease of the breast and usually shows favorable biology, with or without invasion. We experienced an SPC with invasion and aggressive biology, and report its characteristic image findings in this article. A 54-year-old woman with breast cancer, histologically diagnosed at another hospital, was admitted to our hospital for a left femoral neck fracture and underwent orthopedic surgery. During the admission, the patient requested that we operate on her breast cancer. Mammography showed a well-circumscribed mass with pleomorphic and linear calcifications, the latter not pointing toward the nipple. Ultrasound clarified many small cystic areas and multiple echogenic spots in the tumor. Magnetic resonance imaging of the mass showed low signal on T1-weighted images, weak high signal on fat-suppressed T2-weighted images, and a slow/plateau pattern on dynamic studies. Core needle biopsy pathologically led to the diagnosis of SPC with invasion. The patient, therefore, underwent a mastectomy and a sentinel node biopsy. Postoperative pathological study showed no lymph node metastasis but aggressive characteristics such as nuclear grade 3, estrogen receptor negativity, and an extremely high Ki-67 labelling index of 80%. The patient, unfortunately, developed skin flap necrosis, had sudden visual deterioration due to the worsening of diabetic retinopathy, and has been unable to receive postoperative adjuvant chemotherapy for more than 3 months. Diagnostic physicians should note that linear calcifications in the mass pointing toward non-nipple directions should be an important diagnostic clue for SPC of the breast. Diagnostic physicians should further note that many small cystic components in the mass are also important diagnostic image findings for SPC of the breast.

## Introduction

Mammography plays the most important role in the diagnosis of breast cancer. Diagnostic physicians judge whether the target lesions are benign or malignant through evaluating the masses and calcifications on mammography. In short, diagnostic physicians generally evaluate tumors to be malignant when they have irregular shapes. Similarly, diagnostic physicians suspect breast lesions to be malignant when having calcifications along with the duct-lobular systems. Conversely, diagnostic physicians evaluate circumscribed round/oval masses and diffuse/regional calcifications to be benign in clinical practice [1].

Next to mammography, ultrasound is a very useful diagnostic modality for breast cancer. Ultrasound is inferior to mammography in that it cannot visualize the entire breast because it only provides tomographic images. Ultrasound, however, has the advantage of low costs, giving no radiation to patients, and being able to clearly visualize masses even in highly dense breasts, where mammography can sometimes hardly depict even large masses [2]. Ultrasound further has the advantage over mammography for being able to clearly depict cystic components in masses.

Dynamic studies of magnetic resonance imaging (MRI) can provide diagnostic physicians with useful information that mammography and ultrasound cannot provide. MRI can further have the advantage over mammography and ultrasound in detecting daughter nodules and ductal spread, leading to optimal selection of surgical options [3].

With or without invasive components, solid papillary carcinoma (SPC) of the breast is a rare disorder in the breast, shows solid growth around inconspicuous and delicate fibrovascular cores located in its center, and often has neuroendocrine differentiation. In addition, the vast majority of SPCs of the breast have estrogen receptor (ER) positivity, i.e., indolent characteristics, and therefore have been generally detected in small sizes [4].

We experienced a large breast SPC with invasion, which had ER negativity, human epidermal growth factor receptor type 2 negativity, and an extremely high Ki-67 labelling index, and herein report its characteristic image findings correlated with the pathological findings.

## Case presentation

**Figure 1:**
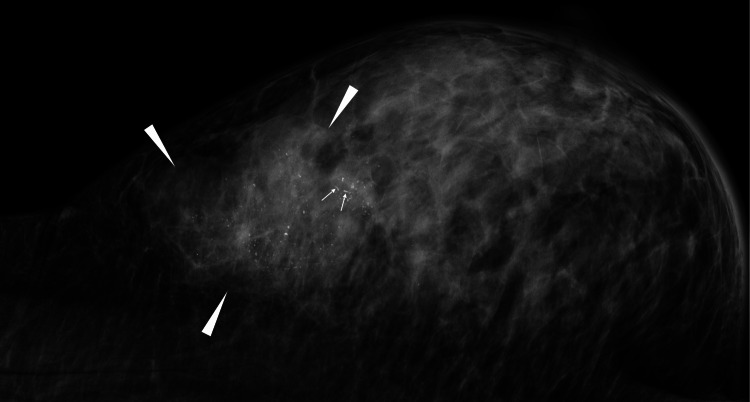
Mammography findings Mammography showed linear calcifications (white arrows) not pointing toward the nipple in the mass (white arrowheads).

A 54-year-old woman with a history of diabetes, diabetes-related right lower limb necrosis, and diabetic retinopathy noticed a large mass in her right breast and visited a hospital, leading to the pathological diagnosis of SPC with invasion by core needle biopsy. Shortly after the pathological diagnosis, the patient unfortunately developed a left femoral neck fracture, was urgently admitted to our hospital, and underwent total hip replacement. Thereafter, the patient requested that we operate on her breast cancer at our hospital. Mammography showed a well-circumscribed mass with pleomorphic and linear calcifications (Figure 1).

**Figure 2:**
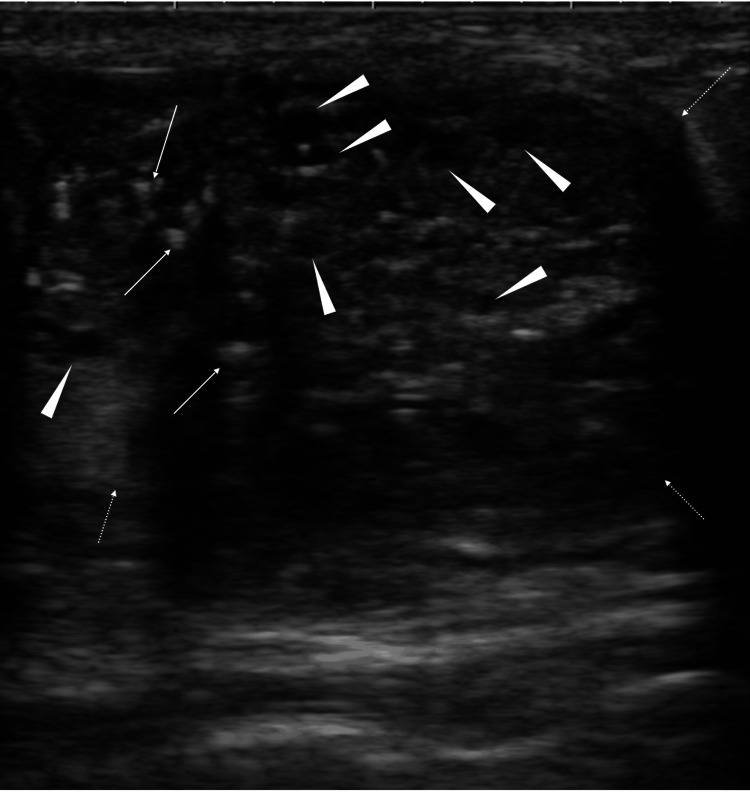
Ultrasound findings Ultrasound showed a well-circumscribed mass (white dotted arrows) with multiple echogenic spots (white arrows) presumably due to the presence of micro calcifications and many small cysts (white arrowheads).

The linear calcifications, however, did not point toward the nipple. Ultrasound further showed many small cystic areas and multiple echogenic spots, i.e., presumable calcifications, in the mass (Figure 2).

**Figure 3:**
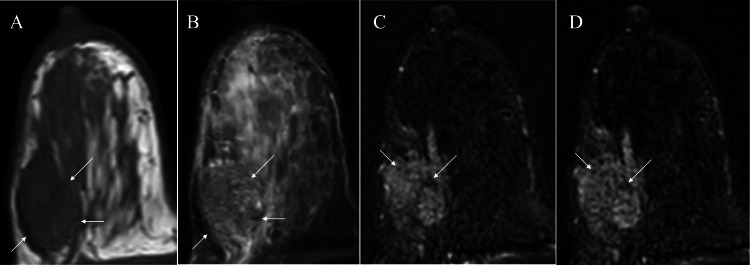
Magnetic resonance imaging (MRI) findings MRI of the tumor showed low signals (white arrows) on T1-weighted images (A), weak high signals (white arrows) on fat-suppressed T2-weighted images (B), slow initial enhancement and non-enhancement areas (white arrows) on early-phase dynamic studies (C), and a plateau pattern and continued non-enhancement areas (white arrows) on delayed-phase dynamic studies (D).

Magnetic resonance imaging of the mass showed low signal on T1-weighted images, weak high signal on fat-suppressed T2-weighted images, and a slow/plateau pattern on dynamic studies (Figure 3).

**Figure 4:**
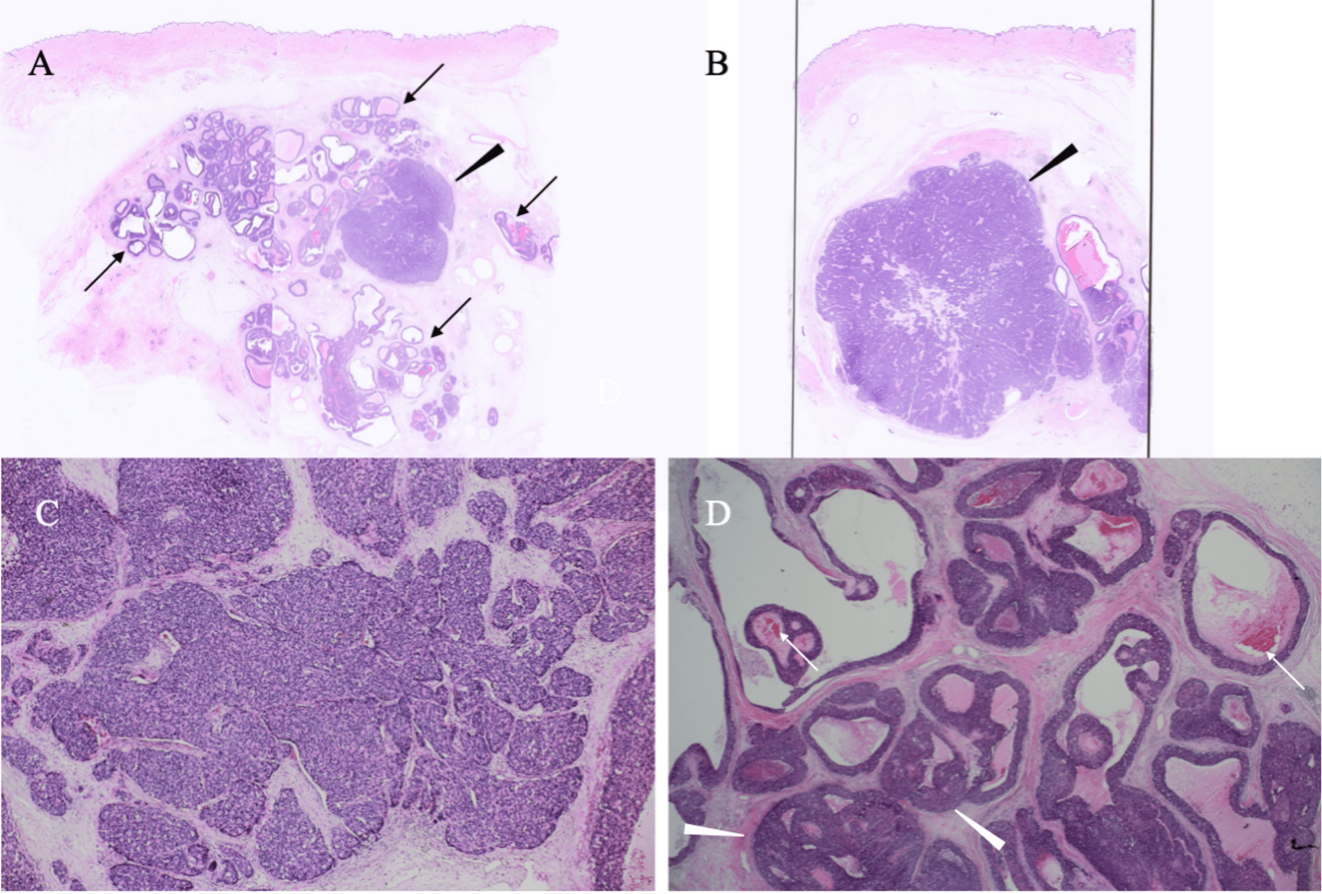
Pathological findings A. Low-magnified view showed predominant non-invasive cancer clusters (black arrows) and an invasive cancer focus 8 mm in size (black arrowhead). B. Low-magnified view showed the larger invasive cancer focus 25 mm in size (black arrowhead). C. Magnified view showed so-called jigsaw structures. D. Magnified view showed micro-calcification (white arrows) in the non-invasive cancer foci growing in a cystic fashion (white arrowheads).

Pathological study of the core needle biopsy specimen showed nuclear grade 3 cancer cells with scant cytoplasm growing in solid and solid/papillary fashions mainly within mammary ducts and focally beyond mammary ducts (Figure 4).

**Figure 5:**
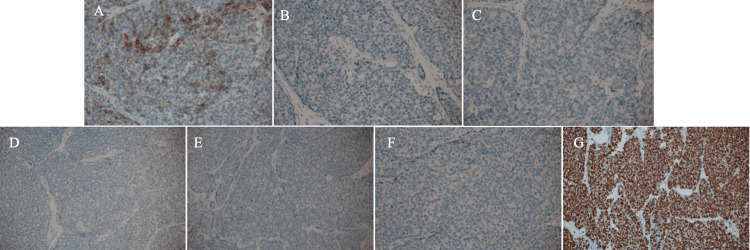
Immunostaining findings Immunostaining showed focal CD56 positivity (A), chromogranin (B), synaptophysin (C), estrogen receptor (D), progesterone receptor (E), human epidermal growth factor receptor type 2 (F) negativity, and a high Ki-67 labelling index of 80% (G).

Immunostaining showed faint ER positivity (Allred score 2), progesterone receptor negativity, human epidermal growth factor receptor type 2 negativity, a Ki-67 labelling index of 80%, AE1/AE3 positivity, E-cadherin positivity, focal CD56 positivity, chromogranin negativity, and synaptophysin negativity, leading to the diagnosis of SPC with invasion (Figure 5).

**Figure 6:**
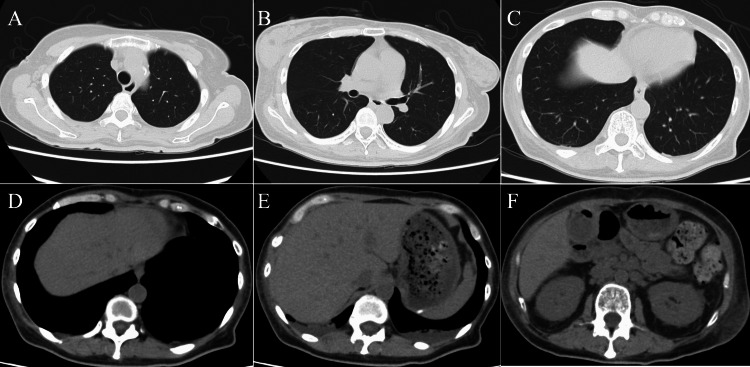
Computed tomography (CT) findings CT showed neither lung (A, upper part; B, middle part; C, lower part) nor liver (D, upper part; E, middle part; F, lower part) metastasis.

Computed tomography (CT) showed neither lung nor liver metastasis (Figure 6).

In addition, no axillary lymph node swelling was judged by palpation, CT, MRI, and ultrasound, which led us to operate on the patientwith mastectomy and sentinel node biopsy, resulting in no lymph node metastasis on frozen section. Postoperative pathological study showed non-invasive SPC in major parts of the tumor with two invasive foci, 25 mm and 8 mm in size, and fibrous stroma around cancer cell clusters. Immunostaining showed that the tumor was a triple-negative phenotype and had a similar Ki-67 labelling index to the core needle biopsy specimen, i.e., 80%. The patient, unfortunately, developed flap necrosis after the operation, has not received any chemotherapies for more than 3 months due to the abrupt onset of diabetes-related severe visual disturbance, and is scheduled for long-term follow-ups on an outpatient basis.

## Discussion

Calcifications suggestive of breast cancer include amorphous, coarse, heterogeneous, fine pleomorphic, and fine linear/linear branching calcifications. Especially, fine pleomorphic or fine linear/linear branching calcifications, only if just being present, strongly suggest breast cancer. In addition, fine pleomorphic or fine linear/linear branching calcifications are the necrotic calcifications in the mammary ducts and, if located in a linear or segmental fashion, directly lead to the diagnosis of breast cancer [1]. In other words, at least fine linear/linear branching calcifications should always point toward the nipple. Fine linear calcifications in this case, however, did not point toward the nipple. This finding can be well explained as follows. The fine linear calcifications appeared in the mammary ducts but had their longitudinal axis originally pointing toward the nipple changed for non-nipple directions by expansive growth of the tumor [4]. Diagnostic physicians, therefore, should strongly suspect SPC when the longitudinal axis of fine linear calcifications does not point toward the nipple.

Diagnostic physicians generally judge intracystic tumors with sharp or dull edges to be benign and malignant, respectively [5]. In invasive ductal carcinomas, cystic parts, which do not have a focal small mass within them but are located in solid masses, generally occupy some parts of the masses as only one or two cystic components and are hardly located in solid masses as many small cystic components. Diagnostic physicians, therefore, should also suspect circumscribed solid masses with multiple small cystic components as SPC in addition to the characteristic calcification findings.

Diagnostic physicians generally detect SPC as ER-positive small masses with favorable biology [6]. This case had neither lymph node metastasis nor distant metastasis, but had a large tumor size, ER negativity, and a high Ki-67 labelling index of 80%. We cannot assume the actual growth rate of this breast cancer because this patient had never undergone screening mammography before noticing a large breast mass. In addition, it remains uncertain why this SPC had aggressive biology. Some studies, however, reported that SPC of the breast had lung and liver metastasis [7,8].

Magnetic resonance imaging of the tumor showed weak high signals on fat-suppressed T2-weighted images and slow initial phase enhancement, many small non-enhanced areas, and a retained enhancement pattern on dynamic studies. As mentioned above, SPC had cancer cells growing in a solid pattern around a fibrovascular stroma [4,5]. Small SPC, therefore, generally shows strong high signals on T2-weighted images due to the abundant cancer cells compared with fibrous components [9]. We judged that weak high signals in the tumor on T2-weighted images were caused by the presence of a large amount of fibrous stroma surrounding the cancer cell clusters. No studies have reported the details of fibrous stroma in SPC to date. Abundant fibrous components in this case might have been correlated with aggressive biology in SPC.

## Conclusions

We encountered an SPC of the breast that had a large tumor size, ER negativity, nuclear grade 3, and a high Ki-67 labelling index of 80%. It naturally remains uncertain why this case had such aggressive characteristics. Diagnostic physicians, however, should note that linear calcifications in the mass pointing toward directions other than the nipple may be an important diagnostic clue for SPC of the breast. Diagnostic physicians should also note that multiple well-demarcated small cystic components within the mass are also important diagnostic imaging findings for SPC of the breast.
